# Composition Dependent Electrical Transport in Si_1−*x*_Ge_*x*_ Nanosheets with Monolithic Single‐Elementary Al Contacts

**DOI:** 10.1002/smll.202204178

**Published:** 2022-09-22

**Authors:** Lukas Wind, Masiar Sistani, Raphael Böckle, Jürgen Smoliner, Lada Vukŭsić, Johannes Aberl, Moritz Brehm, Peter Schweizer, Xavier Maeder, Johann Michler, Frank Fournel, Jean‐Michel Hartmann, Walter M. Weber

**Affiliations:** ^1^ Institute of Solid State Electronics Technische Universität Wien Gußhausstraße 25‐25a Vienna 1040 Austria; ^2^ Institute of Semiconductor and Solid State Physics Johannes Kepler University Altenberger Straße 69 Linz 4040 Austria; ^3^ Swiss Federal Laboratories for Materials Science and Technology Laboratory for Mechanics of Materials and Nanostructures Feuerwerkstrasse 39 Thun 3602 Switzerland; ^4^ CEA‐LETI University Grenoble Alpes 17 Avenue des Martyrs Grenoble 38000 France

**Keywords:** aluminum, germanium, metal‐semiconductor heterostructures, schottky barrier field‐effect transistors, silicon

## Abstract

Si_1−*x*_Ge_*x*_ is a key material in modern complementary metal‐oxide‐semiconductor and bipolar devices. However, despite considerable efforts in metal‐silicide and ‐germanide compound material systems, reliability concerns have so far hindered the implementation of metal‐Si_1−*x*_Ge_*x*_ junctions that are vital for diverse emerging “More than Moore” and quantum computing paradigms. In this respect, the systematic structural and electronic properties of Al‐Si_1−*x*_Ge_*x*_ heterostructures, obtained from a thermally induced exchange between ultra‐thin Si_1−*x*_Ge_*x*_ nanosheets and Al layers are reported. Remarkably, no intermetallic phases are found after the exchange process. Instead, abrupt, flat, and void‐free junctions of high structural quality can be obtained. Interestingly, ultra‐thin interfacial Si layers are formed between the metal and Si_1−*x*_Ge_*x*_ segments, explaining the morphologic stability. Integrated into omega‐gated Schottky barrier transistors with the channel length being defined by the selective transformation of Si_1−*x*_Ge_*x*_ into single‐elementary Al leads, a detailed analysis of the transport is conducted. In this respect, a report on a highly versatile platform with Si_1−*x*_Ge_*x*_ composition‐dependent properties ranging from highly transparent contacts to distinct Schottky barriers is provided. Most notably, the presented abrupt, robust, and reliable metal‐Si_1−*x*_Ge_*x*_ junctions can open up new device implementations for different types of emerging nanoelectronic, optoelectronic, and quantum devices.

## Graphical abstract

The authors report monolithic and single‐crystal heterostructures with abrupt junctions, obtained from a thermal Al‐Si_1−*x*_Ge_*x*_ exchange reaction. Integrated into Schottky barrier transistors with single‐elementary Al contacts, Si_1−*x*_Ge_*x*_ composition‐dependent properties ranging from highly transparent contacts to distinct Schottky barriers are found. The obtained metal‐Si_1−*x*_Ge_*x*_ heterostructures can be a key building block for emerging nanoelectronic, optoelectronic and quantum devices.

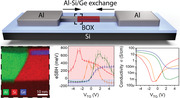

## 1 Introduction

Over the last decades, the integration of Si_1−*x*_Ge_*x*_ and Ge on Si have allowed for the realization of high‐speed heterojunction bipolar transistors. More recently, Si_1−*x*_Ge_*x*_ materials have found application as active regions and raised epitaxial source and drain electrodes in highly scaled p‐channel field‐effect transistors (FETs) for the realization of very‐large‐scale integration (VLSI) systems.^[^ ^1^ ^]^ Despite these efforts, the continuous scaling of metal–oxide–semiconductor field‐effect transistors (MOSFETs) is approaching physical limits where the nature of deterministic charge carrier separation between source and drain by an energy barrier is not applicable anymore.^[^ ^2^, ^3^ ^]^ In the quest of overcoming scaling limitations, new lines of research arose. Device research has shifted toward new architectures, materials, and technologies to enable “More than Moore” paradigms,^[^ ^4^ ^]^ extending the mature Si complementary metal‐oxide‐semiconductor (CMOS) platform.^[^ ^5^ ^]^ In this regard, Si_1−*x*_Ge_*x*_ and Ge active regions integrated on Si platforms are promising candidates for future optoelectronic devices^[^ ^6^ ^]^ and the realization of energy efficient steep subthreshold switches such as band‐to‐band tunneling transistors (TFETs),^[^ ^7^, ^8^ ^]^ negative capacitance Ge nanowire FETs,^[^ ^9^, ^10^ ^]^ and positive feedback FETs.^[^ ^11^ ^]^ Conventionally, degenerately doped semiconductor regions in combination with thin layers made of transition‐metal semiconductor alloys, such as metal‐silicides^[^ ^12^ ^]^ and metal‐germanides,^[^ ^13^ ^]^ have been used to obtain ohmic contacts to most Si_1−*x*_Ge_*x*_ and Ge based devices. Toward the achievement of ohmic contacts, pinning‐free metal semiconductor contacts have been explored in Si and Ge through the use of thin insulator interlayers, such as thermal Si_3_N_4_,^[^ ^14^ ^]^ titania,^[^ ^15^ ^]^ and Ni‐oxide^[^ ^16^ ^]^ interlayers amongst others. Lately, the use of Bismuth contacts having a comparatively low electron density, have successfully shown to minimize pinning effects in Si and Ge.^[^ ^17^ ^]^ Nevertheless, as dimensions of semiconductor devices scale down, precise dopant control and thus contact properties get affected by statistical variability in dopant concentration.^[^ ^18^ ^]^ Additionally, surface depletion effects and even the dielectric mismatch between the semiconductor region and the surrounding insulator induce severe problems.^[^ ^19^ ^]^ To address these issues, diverse emerging electronic device concepts consider metallic junctions providing the functional diversification of transistors.^[^ ^20^, ^21^ ^]^ Thereto, device concepts include Schottky barrier field effect transistors (SBFETs) with low or even negligible barrier heights, either achieved through dopant segregation^[^ ^22^, ^23^ ^]^ or the use of ultrathin insulator depinning interlayers^[^ ^14^, ^15^, ^16^ ^]^ as well as the selective control of charge carrier type and concentration in so called reconfigurable FETs (RFETs).^[^ ^24^, ^25^, ^26^, ^27^ ^]^ The later are capable to overcome the static nature of CMOS by runtime reconfiguration of the transistor, that is, by programming the predominant charge carrier type. Beyond the use in emerging nanoelectronic devices, Si_1−*x*_Ge_*x*_ and Ge further offer an inherently strong spin‐orbit coupling and the ability to host superconducting pairing correlations, providing a high potential for encoding, processing, or transmitting quantum information.^[^ ^28^ ^]^ Consequently, integrated into hybrid superconductor–semiconductor devices, such as a Josephson field‐effect transistors, gate‐tunable superconducting qubits could be realized.^[^ ^28^ ^]^ Thereto, the use of highly transparent superconducting contacts is essential.^[^ ^29^ ^]^ Irrespective of the field of application, reproducible, void‐free, and reliable electrical contacts with defined properties and contact area are highly important. Moreover, the strength of the Fermi level pinning, the related contact resistivities and the yield of functional devices typically depend on the actual stoichiometry and crystallographic phase and interface orientation of the metallic material having intimate contact with the semiconductor according to the metal induced gap states (MIGS)^[^ ^30^ ^]^ and chemical bonding theories.^[^ ^31^ ^]^ To overcome the difficulty in reproducibility and in the deterministic definition of the metal phase in metal‐Si/Ge heterostructures,^[^ ^32^ ^]^ contacts composed of single‐element metals are a highly rewarding strategy for diverse next‐generation nanoelectronic, optoelectronic, and quantum devices^[^ ^33^, ^34^, ^35^, ^36^ ^]^ as well as for providing strategies for pinning‐free contacts as shown recently with Bi/Si and Bi/Ge contact systems.^[^ ^17^ ^]^ Despite a vast variety of different nanoelectronic,^[^ ^37^, ^38^ ^]^ optoelectronic,^[^ ^39^, ^40^ ^]^ and superconductor–semiconductor hybrid devices^[^ ^41^, ^42^, ^43^ ^]^ based on Si_1−*x*_Ge_*x*_ layers of various compositions, a systematic investigation of the structural and electronic properties of Al‐Si_1−*x*_Ge_*x*_ heterostructures obtained from a thermally induced Al‐Si_1−*x*_Ge_*x*_ exchange is still not available. In this respect, the work at hand discusses the Si_1−*x*_Ge_*x*_ composition‐dependent properties of SBFETs based on monolithic and single‐crystalline heterostructures with abrupt and flat junctions. The thereof obtained Si_1−*x*_Ge_*x*_ devices reveal distinctly different injection capabilities ranging from highly transparent contacts to distinct Schottky barriers for electrons and holes, that could be a key building block for emerging nanoelectronic, optoelectronic, and quantum devices.

## 2 Results and Discussion

**Figure 1:**
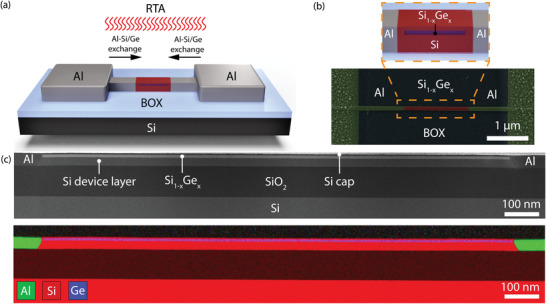
a) Schematic illustration of the Al‐Si_1−*x*_Ge_*x*_‐Al heterostructure after the thermally induced Al‐Si_1−*x*_Ge_*x*_ exchange. b) False‐color SEM image showing an actual device. The zoomed‐in view shows a schematic of the epitaxially grown Si‐Si_1−*x*_Ge_*x*_‐Si stack monolithically embedded in the proposed metal–semiconductor heterostructure. c) Overview STEM image and EDX map of the entire axial Al‐Si_1−*x*_Ge_*x*_‐Al heterostructure monolithically integrated with Al contacts.

In this paper, we report on the systematic investigation of a thermally induced Al‐Si_1−*x*_Ge_*x*_ exchange in nanosheets patterned from nominally undoped Si_1−*x*_Ge_*x*_ layers of different stoichiometric composition that were epitaxially grown on silicon on insulator (SOI) wafers. Such stacks for active regions are highly relevant for the diverse emerging nanoelectronic devices discussed in the Introduction. A high‐resolution scanning transmission electron microscopy (HRSTEM) image of an epitaxially grown vertical Si‐Si_1−*x*_Ge_*x*_‐Si stack is shown in Figure S1, Supporting Information. To demonstrate the potential of this approach and material system, we systematically investigate the electrical properties of Al‐Si_1−*x*_Ge_*x*_‐Al heterostructures based on omega‐gated SBFETs. The presented study focuses on a monolithic contact formation via a thermally induced exchange reaction between the Si and Si_1−*x*_Ge_*x*_ layers and Al contact pads, carried out by rapid thermal annealing (RTA) at *T* = 774 K. In comparison to other metals, as for example, Ni, Pt, Co, or Cu, Al does not form any intermetallic phases.^[^ ^32^ ^]^ Instead, single‐elementary Al contacts to Si/Ge are obtained.^[^ ^36^, ^44^ ^]^ Moreover, the Al‐Si_1−*x*_Ge_*x*_ material system retains its elementary composition even in the event of applying subsequent annealing steps, thus allowing for the formation of reliable and abrupt metal–semiconductor junctions. In contrast, other metals tend to form various temperature‐ as well as crystal orientation dependent silicide or germanide alloy phases within the finally obtained structure.^[^ ^32^, ^45^ ^]^ **Figure** **1**a shows a schematic illustration of the obtained heterostructure after RTA, enabling a monolithic integration of the epitaxially grown Si_1−*x*_Ge_*x*_ stack into axially extended metal–semiconductor heterostructures (see magnified view in the inset of Figure 1b). The false‐color SEM image in Figure 1b shows dark segments (colored in green) extending from the Al contact pads, which prolonged within the Si_1−*x*_Ge_*x*_ nanosheet (colored in red) during annealing. The formation of this metal–semiconductor heterostructure can be understood by examining the phase diagram and the diffusion behavior of the Al–Ge^[^ ^46^ ^]^ and Al–Si^[^ ^47^ ^]^ material system for the applied RTA process at *T* = 774 K (see Table S1, Supporting Information). Whereas the diffusion coefficients of Ge and Si in Al as well as the Al self‐diffusion (Al in Al) are comparatively high, the diffusion coefficients of Al in Ge^[^ ^48^, ^49^ ^]^ and Si^[^ ^50^, ^51^ ^]^ are several orders of magnitude smaller. According to this distinct asymmetry, Al is effectively supplied via fast self‐diffusion from the Al source and released to the Si_1−*x*_Ge_*x*_ nanosheet to compensate Si_1−*x*_Ge_*x*_ outdiffusion (see Table S1 and Figures S2 and S3, Supporting Information). Provided that Si_1−*x*_Ge_*x*_ diffusion in Al takes place through interstitials,^[^ ^52^ ^]^ it is assumed that Si and Ge atoms can diffuse across the entirely exchanged Al segment and ultimately through the Al pads and/or to the structure surface, depending on the available surface passivation. Considering the relatively low annealing temperature of *T* = 774 K and the short annealing duration (≤5 min), the diffusion of Ge in Si and vice versa should be negligible.^[^ ^53^ ^]^ Interestingly, extended annealing resulted in the full nanosheet being transformed into pure Al, resulting in a resistivity of ρ = (9.7 ± 4.4) × 10^−8^ Ω m. Because of an increased influence of surface scattering in nanostructures,^[^ ^54^ ^]^ the obtained resistivity is ≈3.5 times larger than that of bulk Al.^[^ ^49^ ^]^ Further, the resistivity of the obtained Al nanosheets from two‐point *I*/*V* measurements as a function of temperature in the range between *T* = 77.5 and 400 K compared to bulk Al can be seen in Figure S4, Supporting Information. In agreement with the decrease of phonon scattering at lower temperatures of metals,^[^ ^55^ ^]^ a decrease of resistivity for lower temperatures is evident. Notably, upon cooling below the transition temperature of Al (*T*_C_ = 1.25K),^[^ ^56^ ^]^ the Al nanosheets were found to be superconducting, which is an essential prerequisite for future superconductor–semiconductor hybrid devices based on the proposed Al‐Si_1−*x*_Ge_*x*_‐Al platform. At room‐temperature, breakdown current densities of *J*_max_ = (8.9 ± 2) × 10^11^ A m^−2^, comparable to that of Ni_*x*_Si_1−*x*_‐Si NWs, were measured.^[^ ^57^ ^]^ Importantly, the conducted measurements of pure Al nanosheets suggest reliable high‐quality metallic contacts with negligible parasitic series resistance to the Si_1−*x*_Ge_*x*_ channel. To reveal the composition of the obtained Al‐Si_1−*x*_Ge_*x*_‐Al heterostructures, Figure 1c depicts an overview cross‐sectional TEM image and energy‐dispersive X‐ray spectroscopy (EDX) map showing the entire axial Si‐Si_1−*x*_Ge_*x*_‐Si heterostructure monolithically integrated with Al contacts. These investigations show the presence of both a distinct Al‐Si as well as an Al‐Si‐Si_1−*x*_Ge_*x*_ interface.

**Figure 2:**
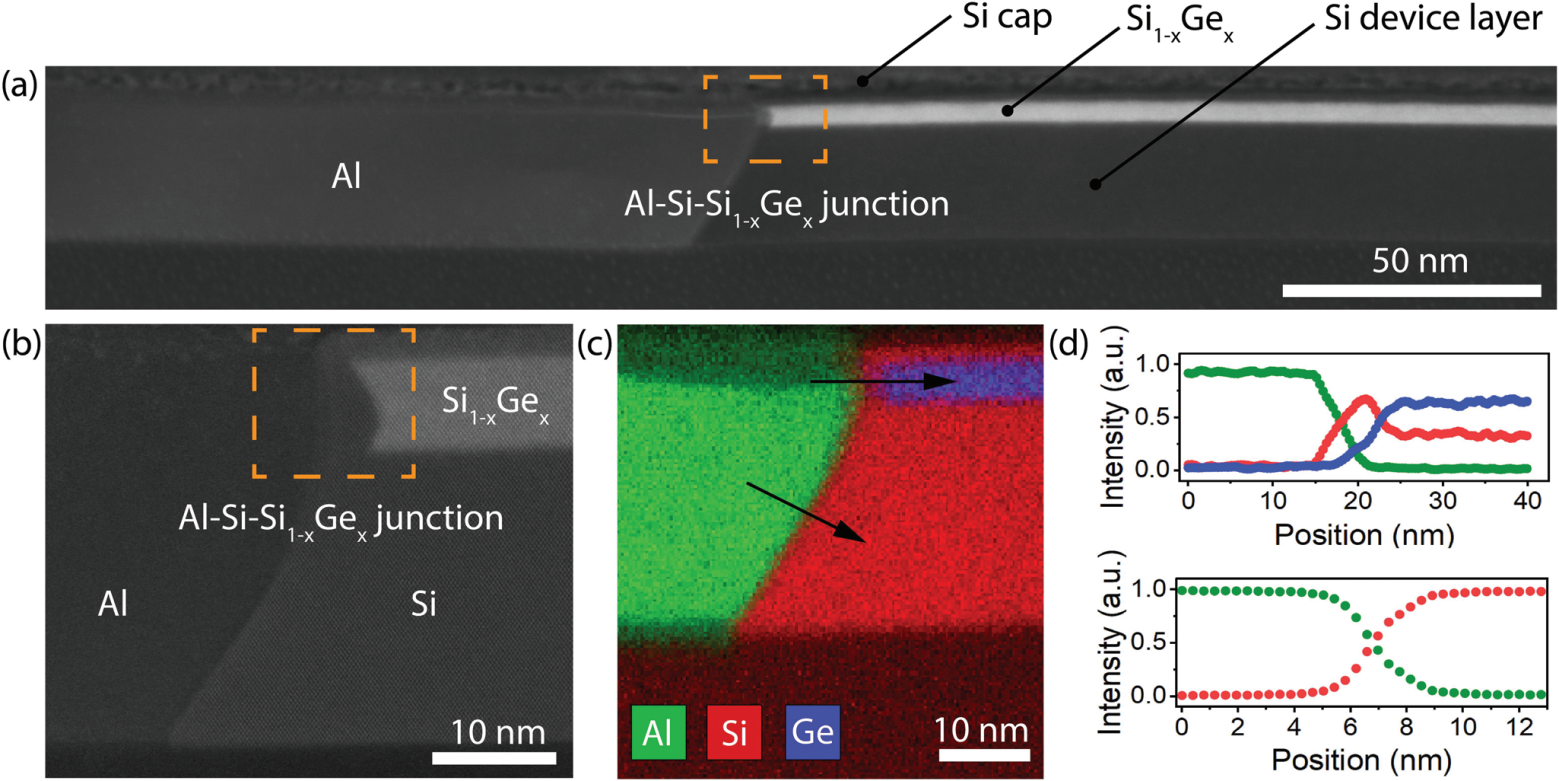
a) Overview HRSTEM image of the entire Al‐Si_1−*x*_Ge_*x*_‐Al heterostructure and b) close‐up view of the metal–semiconductor interface. c) EDX map of the Al‐Si‐Si_1−*x*_Ge_*x*_ junction and d) respective EDX linescan across the abrupt Al‐Si‐Si_1−*x*_Ge_*x*_ and Al‐Si interface. The color coding is carried over from Figure 2c.

Next, we conducted detailed investigations by TEM and EDX of all Si_1−*x*_Ge_*x*_ compositions to better understand the structural properties and the elemental composition of the obtained Al‐Si_1−*x*_Ge_*x*_‐Al heterostructures. Exemplary for the Al‐Si_1−*x*_Ge_*x*_ diffusion behavior, the following discussion is based on the sample with a Si_0.25_Ge_0.75_ layer in vertical Si‐Si_1−*x*_Ge_*x*_‐Si arrangement monolithically embedded between two Al contacts. Thereto, a special focus was set on the Al‐Si_1−*x*_Ge_*x*_ interface. As shown in **Figure** **2**a and representative for all investigated Si_1−*x*_Ge_*x*_ compositions, a double interface between the Al contacts obtained from the exchange reaction and the apparently pristine Si_1−*x*_Ge_*x*_ region was found. In‐between the Al reacted part and the unreacted Si_1−*x*_Ge_*x*_ an Al‐Si‐Si_1−*x*_Ge_*x*_ multi‐heterojunction is formed, which is indicated in the orange box of Figure 2b. This is in agreement with previous investigations of the Al diffusion in Si_0.67_Ge_0.33_ nanowires.^[^ ^58^ ^]^ Remarkably, utilizing this thermal exchange mechanism, no grain boundaries as well as lattice mismatches are observed. Moreover, using molecular beam epitaxy (MBE)‐grown layers, featuring pronounced stacking capabilities (see Experimental Section) no lattice mismatches between the semiconducting layers are observed. Interestingly, independent of the Si_1−*x*_Ge_*x*_ composition, the Al‐Si‐Si_1−*x*_Ge_*x*_ double interface was evident. As also the pure Ge layer showed this junction, it is most likely that the Si‐rich segment is related to the presence of the Si device layer below the Si_1−*x*_Ge_*x*_ layer. This finding is in contrast to the aforementioned investigations on the Si_0.67_Ge_0.33_ nanowires, where the Si‐rich region between the reacted and unreacted part of the nanowire was assumed to be a result of the Al‐Si_1−*x*_Ge_*x*_ diffusion dynamics.^[^ ^58^ ^]^ To entirely clarify the origin of the formed axial Al‐Si‐Si_1−*x*_Ge_*x*_ multi‐heterojunction and gain more detailed insights regarding the diffusion dynamics, temperature‐dependent in situ TEM heating experiments would be necessary, which is a very complex task involving the heating of a cross‐sectional TEM lamella. With respect to the crystal structure, the remaining Si_1−*x*_Ge_*x*_ segment showed a diamond structure, while the intruded Al contact was identified as a face‐centered cubic structure. The Si/Si_1−*x*_Ge_*x*_ is oriented in a [110]‐zone axis with a 001 out‐of‐plane orientation. The interface between Al and Si follows a {111} facet of Si for the silicon dominated part and curves toward a {110} facet close to the Si_1−*x*_Ge_*x*_ containing channel. This channel itself is terminated by two {111} facets bordering the Si interlayer. Notably, regarding reliability and reproducibility, along the entire investigated Al‐Si_1−*x*_Ge_*x*_‐Al heterostructures, the TEM analysis showed no signs of void‐formation, which are common for bulk Al‐Si_1−*x*_Ge_*x*_ junctions. Besides the excellent Al‐Si_1−*x*_Ge_*x*_ interface quality, the proposed thermally induced Al‐Si_1−*x*_Ge_*x*_ exchange overcomes the complex growth kinetics of common Ni_*x*_Si_1−*x*_‐Si_1−*x*_Ge_*x*_ contacts, which exhibit strong variability and yield issues.^[^ ^59^ ^]^ Further, to obtain a more precise chemical characterization, Figure 2c shows the EDX quantification of the Al‐Si‐Si_1−*x*_Ge_*x*_ interface. A complete replacement of the Si_1−*x*_Ge_*x*_ layer by the Al during the thermal exchange reaction, without any Al contamination in the unreacted Si_1−*x*_Ge_*x*_ segment was detected within the resolution limit of the EDX (<1%) measurement equipment. This assumption is in agreement with the supercurrent measurement mentioned above of the entirely exchanged region below the critical current of Al, as this would only be expected for pure Al layers. Figure 2d shows EDX linescans along the Al‐Si‐Si_1−*x*_Ge_*x*_ as well as the Al‐Si interface, revealing a sharply defined Si‐rich segment sandwiched between the intruded Al contact and the unreacted Si_1−*x*_Ge_*x*_ segment of ≈ 5 nm as well as an abrupt Al contact to the Si device layer.

**Figure 3:**
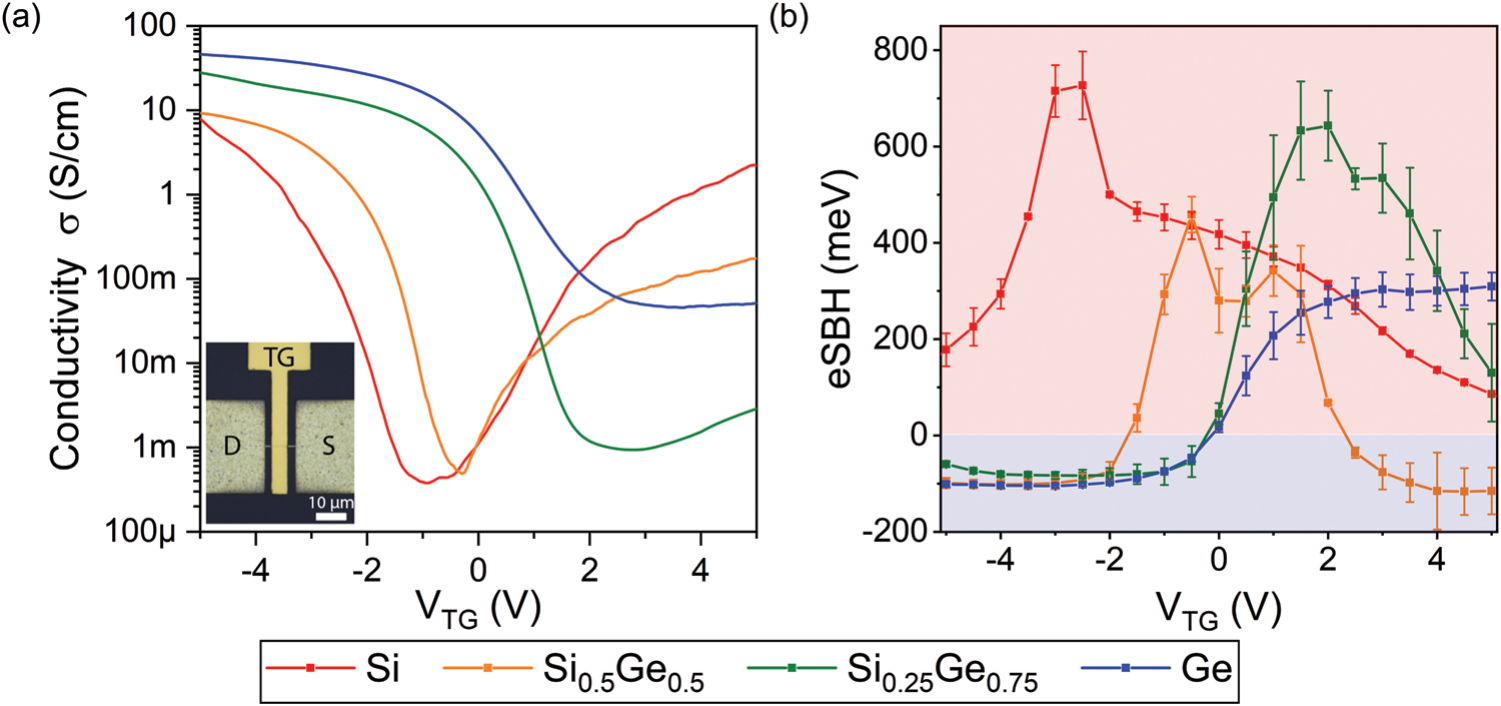
a) Gate‐dependent conductivity of top‐gated Si_1−*x*_Ge_*x*_ based devices for a bias voltage of *V*_D_ = 1 V. The inset shows a microscope image of a top‐gated Al‐Si_1−*x*_Ge_*x*_‐Al heterostructure. b) Effective energy barrier as a function of the top‐gate voltage *V*_TG_, obtained from the slope of the activation energy plot of ln(*J*/*T* ^2^) versus 1000/*T* for various Al‐Si_1−*x*_Ge_*x*_‐Al heterostructures. The gate‐tunability is visualized by the shading, which differentiates between distinct Schottky barriers (red background) and highly transparent contacts (blue background).

To discuss the electrical characteristics of the obtained Al‐Si_1−*x*_Ge_*x*_‐Al nanosheets and their respective contact properties across their multi‐heterojunctions, mesa structures were patterned and omega‐shaped top‐gates were fabricated atop, encompassing a 10 nm thick Al_2_O_3_ gate‐dielectric (see inset of **Figure** **3**a) and Ti/Au electrodes. To achieve devices with a channel length of *L* = 1 µm, consecutive thermal annealing steps accompanied by SEM imaging were applied. This is enabled by the nature of the Al‐Si_1−*x*_Ge_*x*_ exchange resulting Si_1−*x*_Ge_*x*_ nanosheets being contacted by single‐elementary Al contacts. In contrast, common metal‐silicides/‐germanides tend to encounter phase changes with each annealing step.^[^ ^12^, ^32^, ^60^ ^]^ Operated as SBFETs and based on the transfer characteristic for applying a bias voltage of *V*_D_ = 1 V, Figure 3a shows the gate‐dependent conductivity of the investigated Si_1−*x*_Ge_*x*_ compositions. Additionally, device performance parameters of the different top‐gated Si_1−*x*_Ge_*x*_ SBFETs are provided in Table S2, Supporting Information, including the conductivities (σ_on_) and subthreshold slopes (SS). We observe a pronounced and relative symmetric ambipolar characteristic with hole‐dominated transport for *V*_TG_ < −1 V and electron‐dominated transport for *V*_TG_ > 0 V for the pure Si sample. However, the ambipolarity is gradually decreasing with increasing the Ge content of the Si_1−*x*_Ge_*x*_ layer, with the pure Ge layer sandwiched by Si revealing pure p‐type behavior. In consequence, it can be concluded that the Si‐rich phase between the Al and Si_1−*x*_Ge_*x*_ does not seem to influence the Fermi level pinning. We believe that this Si interlayer is too thin and thus dominant tunneling practically leaves changes in the Fermi level pinning unaffected. This behavior can be understood considering the strong Fermi level pinning of Al close to the valence band of Ge,^[^ ^61^ ^]^ leading to a dominant p‐type conduction. In contrast, Al‐Si junctions show mid‐gap Fermi level pinning, resulting in an ambipolar transfer characteristic.^[^ ^44^, ^62^ ^]^ In this respect, approaches to tune the strength of the Fermi level pinning were already published, as for example, introducing (nitride‐)interlayers^[^ ^14^ ^]^ or layers of carbon nanotubes^[^ ^63^ ^]^ between the metal and semiconductor, utilizing different passivations^[^ ^64^, ^65^ ^]^ or van der Waals stacking approaches,^[^ ^66^, ^67^ ^]^ leading to a reduction of the tunneling barrier thickness. Coinciding with a gradual increase of the on‐current in p‐mode (*V*_TG_ = −5 V), due to a lower band‐gap with increasing Ge content and larger carrier concentration, the conductivity of the intrinsic point (point of lowest conductivity) is increasing by orders of magnitude and is shifted to higher gate‐voltages for increasing Ge content. Assuming thermionic emission, the so called effective Schottky barrier height (eSBH) for various Si_1−*x*_Ge_*x*_ compositions ranging from pure Si to pure Ge nanosheets embedded in Al‐Si_1−*x*_Ge_*x*_‐Al heterostructures was obtained from the respective slope of the activation energy plot of ln(*J*/*T* ^2^) versus 1000/*T* (see Figures S5–S7, Supporting Information). Analyzing the eSBH allows to investigate the symmetry of the transfer characteristic of the proposed device architectures, and additionally gives an experimental approach to quantitatively describe the dominant injection properties of charge carriers into the semiconducting channel. While the used model allows to show the tunability of the barrier injection, it needs to be considered that it does not allow to unequivocally differentiate between thermionic and tunneling injection, as the total current is taken into account for the evaluation of the eSBH. In this respect, the injection mechanism depends on the top‐gate voltage *V*_TG_ and thus the eSBH is evaluated in dependence of the gate voltage. In this respect, the total effective activation energy is evaluated in dependence of the gate voltage. Except for the pure Si sample, all Si_1−*x*_Ge_*x*_ stacks showed the Al‐Si‐Si_1−*x*_Ge_*x*_ double interface, which distinctly differs from the gate‐dependent eSBHs. In good accordance with the gate‐dependent conductivity shown in Figure 3a, the pure Si sample showed two relatively symmetric eSBHs for holes and electrons. Interestingly, the exact opposite is observed for increasing the Ge content to 50%, revealing transparent contacts for both electrons and holes, which is indicated by a negative activation energy for −2 V ≤ *V*_TG_ ≤ 2 V. Thus, it is evident that tunneling through a very thin barrier dominates transport resulting in a small contact resistance. Additionally, it needs to be considered that the Fermi level still pins close to the valence band, which also affects the n‐type regime (*V*_TG_ > 0 V), leading to negative eSBH values and in consequence could indicate efficient transport through the hole gas within the Si_1−*x*_Ge_*x*_ layer confined within the Si.^[^ ^68^ ^]^ Nevertheless, the conductivity of the Si_0.5_Ge_0.5_ (cf. Figure 3a) is lower in comparison to that of pure Si, as electrons still face a higher barrier than in pure Si. While for a further increase of the Ge content by 25% (Si_0.25_Ge_0.75_) the high transparency of holes remained, the barrier for electrons is clearly increasing. Complementing the presented study, the pure Ge layer revealed distinct p‐type behavior with a highly transparent contact for holes. This behavior can be understood, considering that the Ge is sandwiched by Si from the top and bottom sides, which results in an abrupt discontinuity of the band structure at the Ge‐Si interfaces. This is expected to induce a band offset of ≈500 meV^[^ ^69^, ^70^ ^]^ causing a constant flow of holes from Si to Ge to maintain a constant chemical potential throughout the arrangement.^[^ ^71^ ^]^ Consequently, the band edges are bent at the interface, resulting in holes being confined in the Ge layer close to the Ge‐Si interface, forming a hole‐gas.^[^ ^72^ ^]^ Thus, sweeping the gate‐voltage, the pure Ge layer sandwiched by Si is only capable of tuning the transparency of the junction rather than enabling electron conduction. The existence of the hole‐gas in the vertically confined Si‐Ge‐Si stacked nanosheet is further approved by comparison with the temperature‐dependent resistivity of a reference structure composed of a Ge on insulator (GeOI) wafer excluding the effects of Si layers. As seen in Figure S8, Supporting Information, the GeOI nanosheet shows a distinct increase of the resistivity, due to charge carrier freeze‐out. In contrast, the vertically confined Si‐Ge‐Si stack reveals only a slight resistivity decrease for lower temperatures, which is in agreement with the presence of a hole‐gas.

**Figure 4:**
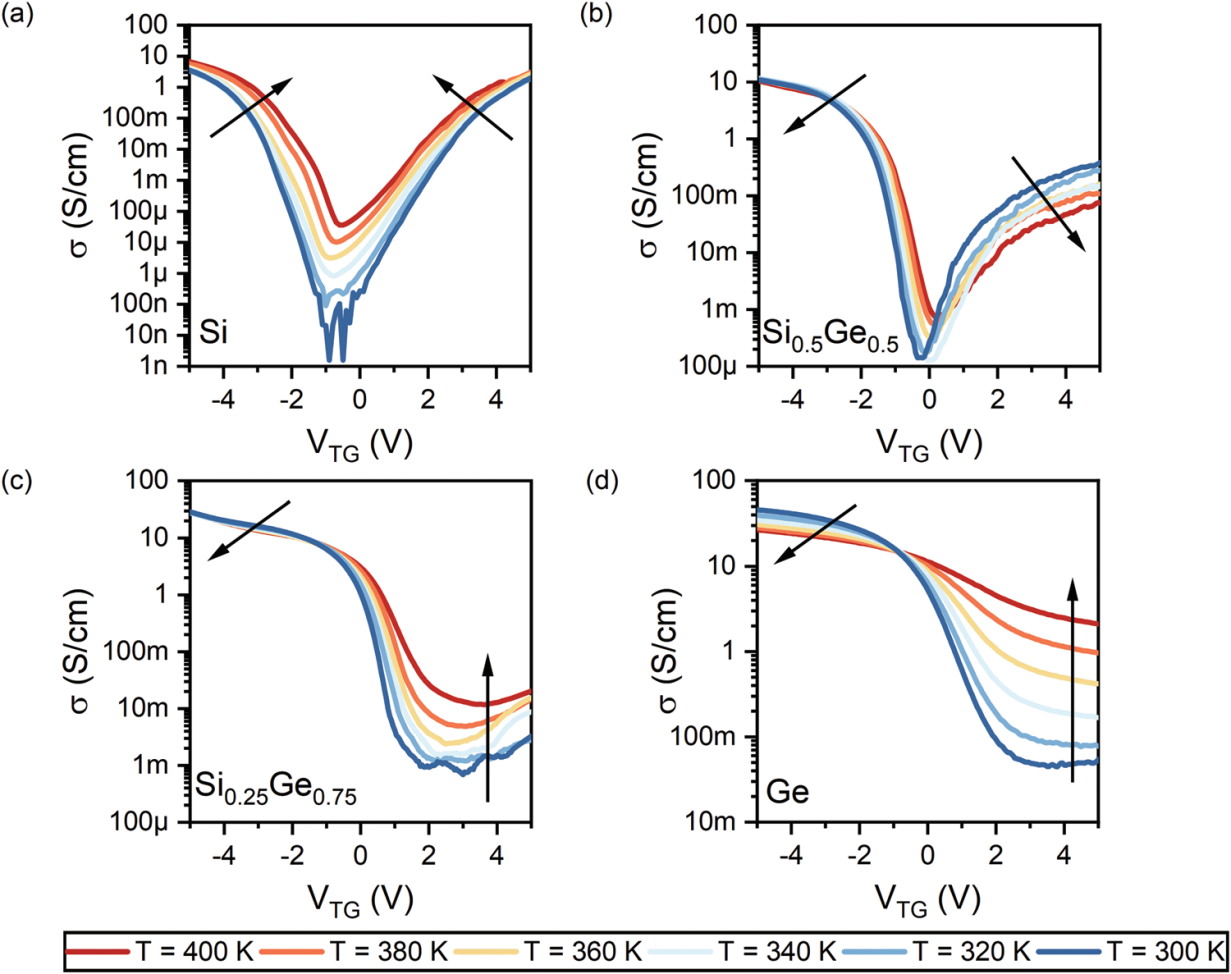
Representative temperature dependent transfer characteristics of a) Si, b) Si_0.5_Ge_0.5_, c) Si_0.25_Ge_0.75_, and d) Ge nanosheets embedded in Al‐Si_1−*x*_Ge_*x*_‐Al heterostructures for a bias voltage of *V*_D_ = 1 V measured in the temperature range between *T* = 300 and 400 K. The arrows indicate either a positive (up) or a negative (down) variation of the current for increasing temperature.

To further investigate the composition‐dependent transport properties of Si_1−*x*_Ge_*x*_ nanosheets embedded in Al‐Si_1−*x*_Ge_*x*_‐Al heterostructures, the temperature dependence of the transfer characteristics for applying *V*_D_ = 1 V between *T* = 300 and 400 K was analyzed. The pure Si sample, shown in **Figure** **4**a, shows an increasing off‐current at elevated temperatures, which can be attributed to thermally generated carriers over the Schottky barrier or a higher rate of injection of charge carriers through thermal assisted tunneling. Further, no substantial increase of the on‐current was observed, which is in agreement with a tunneling‐dominated charge injection. Such a Al‐Si‐Al material system is highly interesting for SBFET‐based RFETs, capable of dynamically reprogramming the operation between n‐ and p‐type even during runtime.^[^ ^73^, ^74^ ^]^ In good agreement with the gate‐dependent eSBH measurement, the Si_0.5_Ge_0.5_ layer showed a negative variation for both hole and electron conduction, indicating the access to two semi‐transparent junction regions (see Figure 4b). Such a system is potentially interesting for SBFET based RFETs with improved on‐currents compared to the Si based systems, as well as for quantum devices that enable the investigation of gate‐tunable charge‐carrier tunneling with both electrons and holes.^[^ ^35^, ^75^, ^76^, ^77^ ^]^ Moreover, in the Si_0.25_Ge_0.75_ layer (see Figure 4c) and even more pronounced for the pure Ge layer confined by Si, (see Figure 4d) an increase of resistance of the p‐mode on‐state (*V*_TG_ ≤ 0 V) was found, indicating that scattering is the main contribution to the resistance at elevated temperatures. This observation is a further indication for tunneling through a thin barrier, which is determining the transport, rather than thermionic emission. Such a highly transparent junction was so far only reported for alternative semiconductor systems such as between carbon nanotubes and Pd contacts^[^ ^78^, ^79^ ^]^ or for the efficient use of Fermi‐level depinning approaches.^[^ ^14^, ^17^ ^]^ In the off‐state of both Si_1−*x*_Ge_*x*_ compositions (*V*_TG_ ≥ 0 V), the resistance decreases with increasing temperature, indicating that thermally activated transport at the contacts is the main contribution to resistance. As the band‐structure of Si_1−*x*_Ge_*x*_ with such high Ge contents (≥70%) is rather closer to the one of Ge than Si,^[^ ^80^ ^]^ and still features electron conduction, the investigated Si_0.25_Ge_0.75_ layer might be interesting for negative differential resistance (NDR) devices based on the electron transfer effect.^[^ ^21^, ^81^, ^82^ ^]^ In contrast, the hole‐gas system based on the pure Ge nanosheet confined by Si, due to the gate‐tunable transparency, could be a key component for quantum computing such as gate‐tunable Josephson junctions, which are an important prerequisite for gatemon or transmon qubits.^[^ ^28^, ^29^ ^]^ Further, the temperature sensitivity of the obtained Al‐Si_1−*x*_Ge_*x*_‐Al nanosheets operated in the off‐regime might be very interesting for the realization of bolometers.^[^ ^40^ ^]^ Thereto, the temperature dependence of the off‐current extracted from Figure 4 for all investigated Si_1−*x*_Ge_*x*_ compositions embedded in top‐gated Al‐Si_1−*x*_Ge_*x*_‐Al heterostructures is shown in Figure S9, Supporting Information. This evaluation suggests that the low off‐current of Al‐Si‐Al heterostructures in combination with the high‐temperature sensitivity would be the preferred system for bolometers. With respect to recent advances in guiding and localizing light at nanoscale, the obtained Al‐Si_1−*x*_Ge_*x*_‐Al heterostructures, due to well‐defined metal–semiconductor junctions and high‐quality Al leads, might also be promising for next‐generation near‐infrared plasmon enhanced optoelectronic devices.^[^ ^39^ ^]^ Thereto, the Al leads would serve as waveguides monolithically connected to a Si_1−*x*_Ge_*x*_ detector, where a plasmon‐driven hot‐electron transfer enhances the photocurrent. Such devices are highly interesting for a vast variety of applications such as boosting the efficiency of energy‐harvesting, photocatalysis, and photodetection.^[^ ^83^ ^]^

## 3 Conclusion

In conclusion, we have methodically investigated the thermally induced Al‐Si_1−*x*_Ge_*x*_ exchange in top‐down fabricated Si_1−*x*_Ge_*x*_ nanosheets based on SOI wafers. Structural investigations by TEM and EDX confirmed the monolithic and single‐crystalline nature of the obtained metal–semiconductor–metal heterostructures. A detailed analysis of the Al‐Si_1−*x*_Ge_*x*_ exchange revealed abrupt and reproducible metal–semiconductor interfaces of high structural quality avoiding the common deficiencies of bulk Al‐Si_1−*x*_Ge_*x*_ junctions. To probe the electrical properties, the proposed Si_1−*x*_Ge_*x*_ nanosheets were integrated into omega‐gated SBFETs. From the gate‐ and temperature‐dependent measurements, it is evident, that Al‐Si_1−*x*_Ge_*x*_‐Al heterostructures constitute a material system with highly tunable properties depending on the Si_1−*x*_Ge_*x*_ composition. Pure Si nanosheets show distinct symmetric eSBHs for both electrons and holes, which are highly interesting for reconfigurable electronics based on RFETs. In contrast, for a Ge content of 50%, two transparent junctions were found, which might be used in quantum devices with gate‐tunable charge‐carrier tunneling for both, electrons and holes. Si_0.25_Ge_0.75_ revealed strongly asymmetric barriers that is, a transparent junction for holes and a distinct eSBH for electrons, that due to a more Ge‐like band‐structure, could be used for NDR based electronics. Finally, due to the vertical Si–Ge–Si stack formed hole‐gas, pure Ge nanosheets provide only hole‐conduction. In this respect, the ability to tune the transparency of the junction using electrostatic gating might enable key components of quantum computing such as gate‐tunable Josephson junctions. Moreover, with respect to photonic applications, the high‐quality Al leads formed to all Si_1−*x*_Ge_*x*_ compositions resemble plasmonic waveguides monolithically embedded with a Si_1−*x*_Ge_*x*_ detector, where a plasmon‐driven hot‐electron transfer should enhance the photocurrent. Most importantly, the high quality of the obtained Si_1−*x*_Ge_*x*_ nanosheets monolithically embedded with single‐elementary Al leads may pave the way for a vast variety of next‐generation nanoelectronic, optoelectronic, and quantum devices that require reliable and reproducible metal–semiconductor–metal heterostructures with abrupt and high‐quality interfaces.

## 4 Experimental Section

### Epitaxial Growth of Si_1−*x*_Ge_*x*_ on SOI

For the growth of the Si_1−*x*_Ge_*x*_ heterostructures on SOI substrates, recent growth strategies for the successful formation of Ge‐rich but pseudomorphic 2D films with low surface roughness deposited on bulk Si substrates were adapted.^[^ ^84^ ^]^ The layers were grown in a Riber SIVA‐45 MBE system on SOI and strained‐silicon‐on‐insulator (sSOI) substrates, both in [100] orientation. The BOX and device layer thickness for the SOI and sSOI samples were 2 µm/30 nm and 130 nm/30 nm, respectively. The Si device layer of the sSOI has an in‐plane lattice constant equal to that of a relaxed Si_0.7_Ge_0.3_ alloy. After a standard substrate cleaning process, the substrates were dipped in hydrofluoric acid (HF 1%) to remove the native oxide before their introduction into the MBE chamber. All substrates were degassed at 973 K for 20 min. For sample G100, grown on sSOI, a 5 nm thick Si buffer layer and a 6 nm Ge layer, were deposited followed by a 2.5 nm thick Si capping layer, all deposited at a growth temperature *T*_G_ = 558 K. Sample G75 received a 10 nm thick Si buffer layer, deposited on SOI substrate at a *T*_G_, ramped from 723 to 823 K, a 6 nm thick Si_0.25_Ge_0.75_ layer at *T*_G_ = 548 K and a 2.5 nm thick Si cap at *T*_G_ = 723 K. Finally, in sample G50, the SOI substrate was covered by a 10 nm Si buffer layer (*T*_G_ ramped from 723 to 823 K), a 5 nm thick Si_0.5_Ge_0.5_ layer, and a 5 nm thick Si capping layer, both deposited at *T*_G_ = 623 K. The lower growth temperatures for layers with higher Ge contents were needed to suppress elastic and plastic strain relaxation.^[^ ^84^ ^]^

### Device Fabrication

The Si_1−*x*_Ge_*x*_ on SOI was patterned using laser lithography and SF_6_‐O_2_ based reactive ion etching. Atomic layer deposition (ALD) was employed to grow a high‐quality ≈10 nm thick Al_2_O_3_ gate‐oxide. Al pads contacting the obtained nanosheets were fabricated by laser lithography, 125 nm Al sputter deposition, preceded by a 15 s BHF dip (7:1) to remove the Al_2_O_3_ at the Al‐Si_1−*x*_Ge_*x*_ contact area and lift‐off techniques. The Al‐Si_1−*x*_Ge_*x*_ exchange reaction was induced by RTA at a temperature of *T* = 774 K in forming gas atmosphere. Omega‐shaped Ti/Au top‐gates were fabricated atop Al–Si_1−*x*_Ge_x_–Al heterostructures, using a combination of electron beam lithography, Ti/Au evaporation (10 nm Ti, 100 nm Au), and lift‐off techniques.

### TEM Measurements

TEM lamella preparation was performed using a Tescan Lyra FIB/SEM. The TEM images were acquired using a Thermo Fisher Scientific Titan Themis 200 G3 outfitted with a SuperX detector used for the EDX maps.

### Electrical Measurements

The electrical measurements as well as the temperature‐dependent measurements were performed using a LakeShore PS‐100 cryogenic probe station and a Keysight B1500A semiconductor analyzer.

## Conflict of Interest

The authors declare no conflict of interest.

## Author Contributions

L.W. and M.S. contributed equally to this work. L.W. and M.S. performed the device fabrication. L.W., R.B. and M.S. conducted the electrical measurements. M.S. wrote the manuscript. J.S. provided expertise on theoretical interpretations of the measured data. P.S., X.M. and J.M. carried out the TEM and EDX measurements and analysis. F.F and J.‐M.H. have fabricated the used sSOI substrates. L.A., J.A. and M.B. designed and grew the samples. M.S and W.M.W. conceived the project and contributed essentially to the experimental design.

## Supporting information

Supporting Information

## Data Availability

The data that support the findings of this study are available from the corresponding author upon reasonable request.
