# Supplementary material for: Composition Dependent Electrical Transport in Si1−x Ge x Nanosheets with Monolithic Single‐Elementary Al Contacts
Source: Small. 2022 Sep 22;18(44):2204178. doi: 10.1002/smll.202204178 (PMC11475588; doi:10.1002/smll.202204178)
Supplement: Supplementary file 1 — Supporting Information [file SMLL-18-2204178-s001.pdf]

NANO small MICRO

Supporting Information

for *Small,* DOI: 10.1002/smll.202204178

Composition Dependent Electrical Transport in $Si_{1-x}Ge_x$ Nanosheets with Monolithic Single-Elementary Al Contacts

*Lukas Wind, Masiar Sistani, Raphael Böckle, Jürgen Smoliner, Lada Vukŭsić, Johannes Aberl, Moritz Brehm, Peter Schweizer, Xavier Maeder, Johann Michler, Frank Fournel, Jean-Michel Hartmann, and Walter M. Weber**

Supporting Information: Composition Dependent Electrical Transport in $Si_{1-x}Ge_x$ Nanosheets with Monolithic Single-Elementary Al Contacts

Lukas Wind,[†] Masiar Sistani,[†] Raphael Böckle,[†] Jürgen Smoliner,[†] Lada Vukǔsić,[‡] Johannes Aberl,[‡] Moritz Brehm,[‡] Peter Schweizer,[¶] Xavier Maeder,[¶] Johann Michler,[¶] Frank Fournel,[§] Jean-Michel Hartmann,[§] and Walter M. Weber[*,†]

†*Institute of Solid State Electronics, Technische Universität Wien, Gußhausstraße 25-25a, 1040 Vienna, Austria*

‡*Institute of Semiconductor and Solid State Physics, Johannes Kepler University, Altenberger Straße 69, 4040 Linz, Austria*

¶*Swiss Federal Laboratories for Materials Science and Technology, Laboratory for Mechanics of Materials and Nanostructures, Feuerwerkstrasse 39, 3602 Thun, Switzerland*

§*University Grenoble Alpes, CEA, LETI, 17 Avenue des Martyrs, 38000 Grenoble, France*

E-mail: walter.weber@tuwien.ac.at

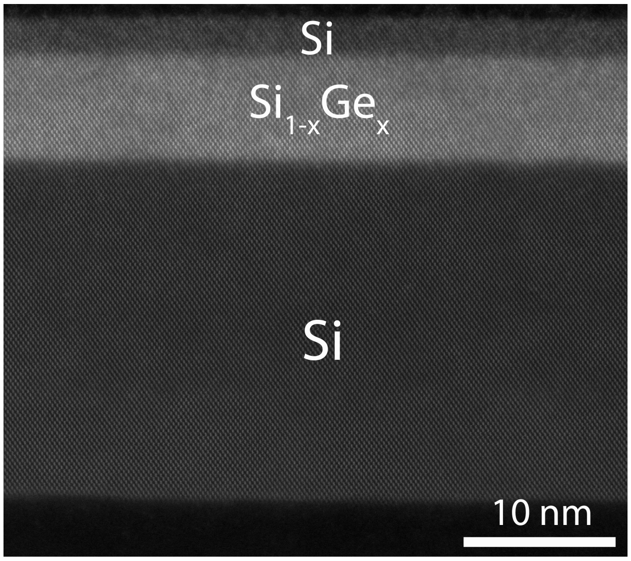

Figure S1: TEM image showing a $Si_{0.25}Ge_{0.75}$ layer embedded in the vertical Si-$Si_{1-x}Ge_x$-Si stack.

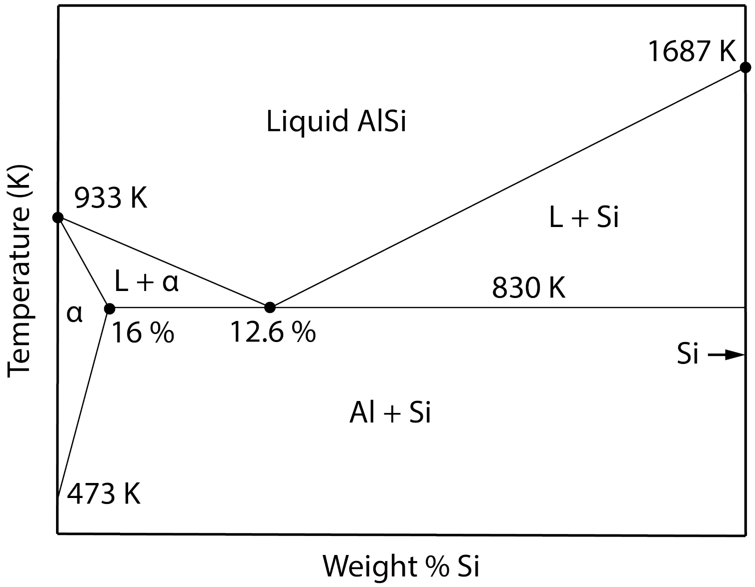

Figure S2: Binary Al-Si phase diagram showing a simple eutectic type with no intermetallic phase formation.[1] The melting points of Al and Si are 993 K and 1684 K, respectively, and the eutectic point is located at a composition of about 12.6 wt % Si with a solid to liquid transition at 830 K.

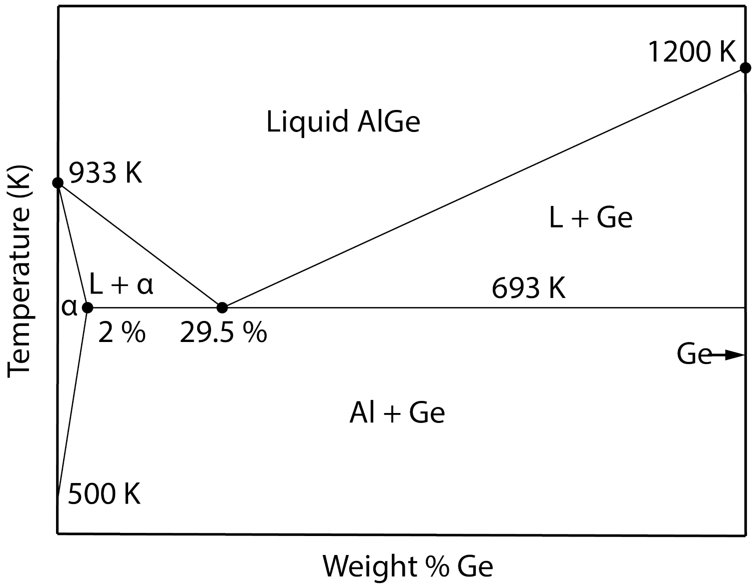

Figure S3: Binary Al-Ge phase diagram showing a simple eutectic type with no intermetallic phase formation.[2] The melting points of Al and Ge are 993 K and 1211 K, respectively, and the eutectic point is located at composition of about 29.5 wt % Ge with a solid to liquid transition at 693 K.

Table S1: Diffusion coefficients of the Al-Si[3,4] and Al-Ge[5,6] material system. The diffusion coefficients are shown for a temperature of T = 774 K.

**Al in Al** ($cm^2\,s^{-1}$)	**Al in Si** ($cm^2\,s^{-1}$)	**Al in Ge** ($cm^2\,s^{-1}$)	**Si in Al** ($cm^2\,s^{-1}$)	**Ge in Al** ($cm^2\,s^{-1}$)	**Si in Si** ($cm^2\,s^{-1}$)	**Ge in Ge** ($cm^2\,s^{-1}$)
6.3×10^{-10}	2.0×10^{-22}	3.3×10^{-20}	4.4×10^{-8}	3.1×10^{-9}	6.5×10^{-19}	8.4×10^{-20}

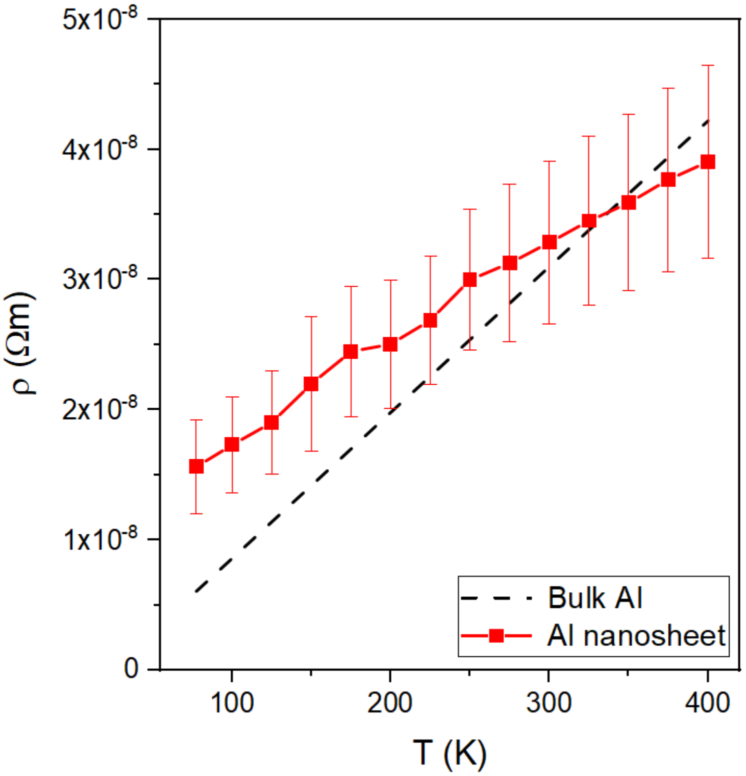

Figure S4: Temperature dependent mean resistivity of five similar Al nanosheets obtained from thermally induced Al-$Si_{1-x}Ge_x$ exchange measured between T = 77.5 K and 400 K. For comparison the temperature dependent resistivity of bulk Al is also plotted (dashed line).

$Si_{1-x}Ge_x$ device performance

For a better comparison of the different $Si_{1-x}Ge_x$ alloys, Table S2 shows the conductivity σ of the on-state as well as the subthreshold slope SS at $V_{TG} = 5\,V$ (n-type) and $V_{TG} = -5\,V$ (p-type). These two parameters are of high importance for the realization of actual devices, as the on-capabilities as well as the transition between the on- and off-state are characteristic parameters for transistors. Note that the conductivity ($\sigma = L/(A \cdot R)$) is chosen as a figure of merit due to the fact, that the applied voltage (and current) as well as the geometries are taken into account, providing a fair comparison between different devices. It needs to be considered that the oxide (thickness) as well as the geometries of the devices are not optimized in the devices presented in this work. Decreasing the oxide thickness, utilizing a high-κ dielectric and reducing the geometrical parameters, would lead to better performance metrics. Additionally, values are stated of a vapor-liquid-solid (VLS)-grown Ge NW contacted by Ni-germanide[7] and a VLS-grown Ge/Si core/shell NW.[8] Note that these devices are ultrascaled with a NW diameter of 18 nm. Remarkably, the Ge/Si core/shell NWs utilize 4 nm of HfO_2 as gate dielectric, further enhancing the performance.

Table S2: Device performance of top-gated vertically stacked $Si_{1-x}Ge_x$ transistors with different Ge concentrations. For the Si-Ge-Si nanosheets no value is given for SS_n, since no electron-conduction is evident. Additionally, VLS-grown Ge as well as VLS-grown Ge/Si core/shell NWs are compared. Note that the Ge/Si core/shell NW[8] utilizes 18 nm thick NWs with a 4 nm thick HfO_2-layer as gate dielectric.

**Vertical device stack**	$\boldsymbol{\sigma_{on,p}}$ (S/cm)	$\boldsymbol{\sigma_{on,n}}$ (S/cm)	$\boldsymbol{SS_p}$ (V/dec)	$\boldsymbol{SS_n}$ (V/dec)
Si device layer	8	2.22	0.50	0.82
Si-$Si_{0.5}Ge_{0.5}$-Si	9.20	0.18	0.35	0.86
Si-$Si_{0.25}Ge_{0.75}$-Si	28	0.003	0.46	3.90
Si-Ge-Si	46.20	0.052	1	-
VLS-grown Ge NW[7]	7.52	0.004	0.83	1.66
VLS-grown Ge/Si core/shell NW[8]	1180	3.93	0.13	0.20

Experimental eSBH modelling

The theoretical assumptions for the experimental eSBH extraction is taken from the work "Metal-Semiconductor Contacts" by E.H. Rhoderick and R.H. Williams.[9] Here, it needs to be considered, that the I/V(-T) approach, which relies on thermionic emission theory, is utilized. In general, this theory is valid for barrier heights larger than $k_B T$ (25.7 meV at T = 300 K) and small bias voltages to avoid barrier lowering, and thus significant tunneling currents. Nevertheless, the contribution of tunneling to the total current plays an important role in the $Si_{1-x}Ge_x$ nanosheets analyzed in the scope of this work. Note that, experimental investigations do not allow to differentiate between thermionic and tunneling contributions to the total current. Moreover, this experimental approximation neglects the potential between the metal and semiconductor and thus leads to a simplified equation. According to E.H. Rhoderick and R.H. Williams the current through the Schottky barrier can be simplified when the applied bias voltage exceeds $3 \times k_B T/q$ (76 mV at T = 300 K). Equation 1 gives the simplified equation based on thermionic emission theory for the evaluation of the total effective activation energy. Note that previous published works promote this model for determining the total effective activation energy.[10,11]

$$J_{TE}(T) = A^* T^2 \exp \frac{-q\phi_{eSBH}}{k_B T} \tag{1}$$

where J_{TE} is the measured current density through the device, A^* is the effective Richardson constant, T is the corresponding temperature and $(q)\phi_{eSBH}$ is the total effective activation energy, which is interpreted as the effective barrier height. Thus, the total effective activation energy can be extracted by measuring the I/V-characteristic at different temperatures and applying the natural logarithm to extract the barrier height of the previous equation. Finally, equation 2 shows the obtained expression. Figure S5 exemplary shows the T-dependent I/V-characteristic of $Si_{0.5}Ge_{0.5}$ device at $V_{TG} = -5$ V.

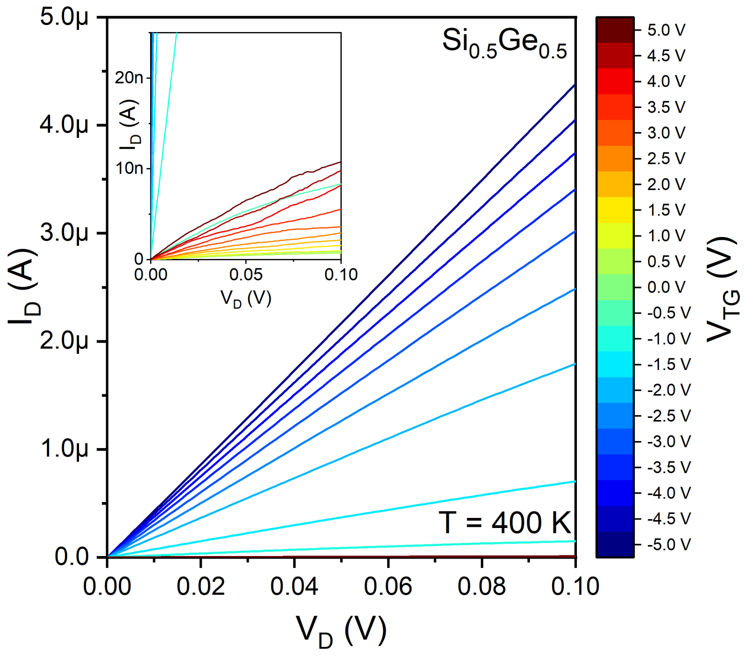

Figure S5: Exemplary temperature dependent I/V-characteristic of a $Si_{0.5}Ge_{0.5}$ based SBFET at $V_{TG} = -5\,V$. As seen in the zoom-in of the I/V in the inset, even a near linear I/V characteristic towards the extrapolated point at $V_D = 0\,V$ was measured.

$$\ln \frac{J_{TE}}{T^2} = \ln A^* - \frac{q\phi_{eSBH}}{k_B T} \tag{2}$$

Thus, by plotting $\ln \frac{J_{TE}}{T^2}$ (y-axis) as a function of $1000/T$ (x-axis), a so-called Richardson plot is obtained (cf. Figure S6).

Using the linear equation and setting the factors of the above equation correspondingly to y = kx + d, the individual parameters can be extracted. By analyzing the slope k, the corresponding $q\phi_{eSBH}$ can be determined for a specific drain bias voltage V_D as depicted in Equation 3.

$$q\phi_{eSBH} = -k \cdot k_B \cdot 1000 \tag{3}$$

where k is the evaluated slope of the Richardson plot. Finally, $q\phi_{eSBH}$ can be plotted over V_D for the evaluation of the total effective activation energy. Extrapolation of the data points to $V_D = 0\,V$ is used to perform a careful estimation of the activation energy then,

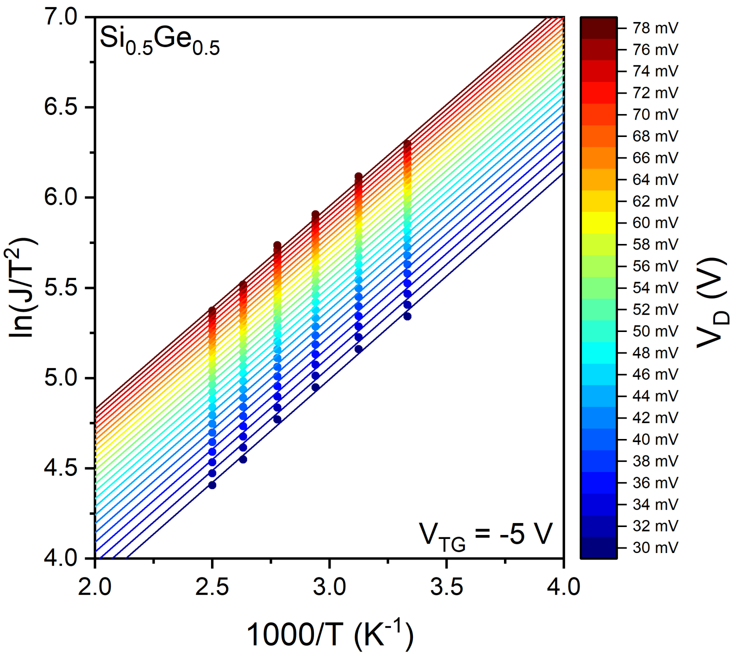

Figure S6: Exemplary $\ln J/T^2$ vs. $1000/T$ (Richardson plot) plot of the $Si_{0.5}Ge_{0.5}$ based SBFET.

including initial effects of barrier lowering and tunneling at the tip of the barrier. Figure S7 shows the corresponding plot at a dedicated $V_{TG} = -5\,V$.

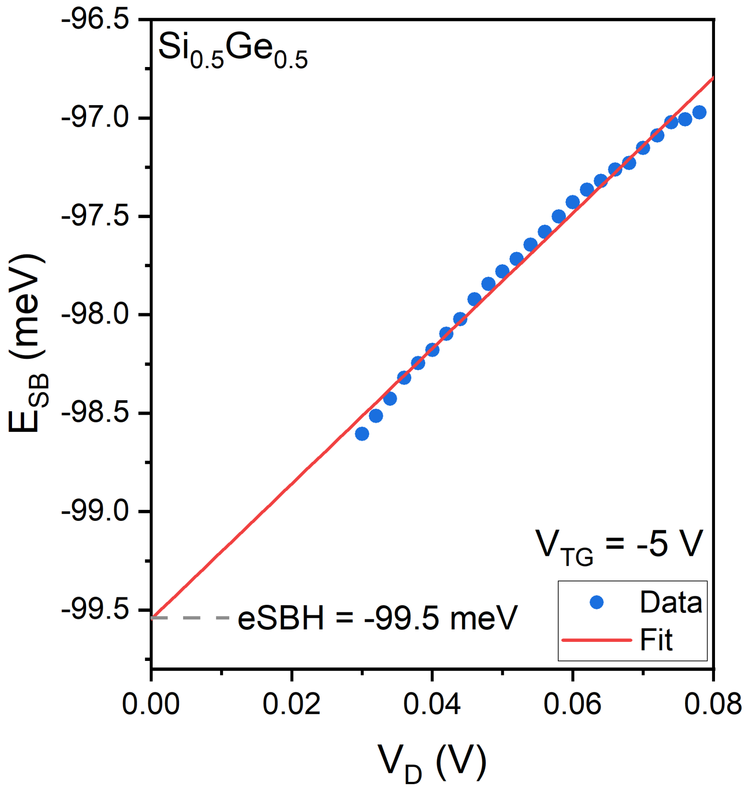

Figure S7: Exemplary eSBH extraction by linear fitting and extrapolating to $V_D = 0\,V$ for $V_{TG} = -5\,V$. In the particular case, the data of the $Si_{0.5}Ge_{0.5}$ based SBFET is shown.

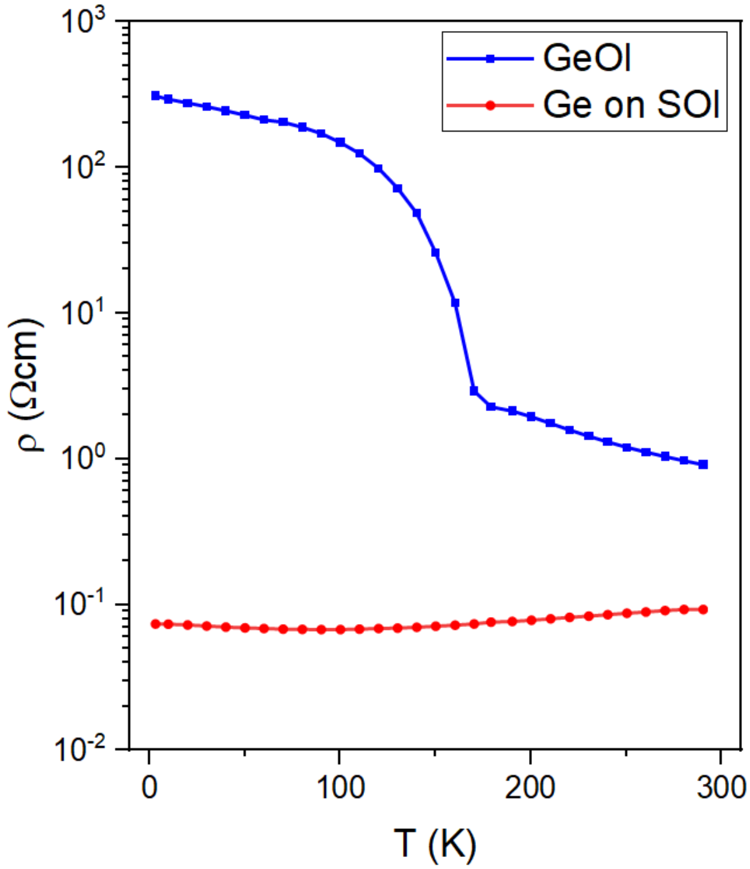

Figure S8: Temperature dependent four-point resistivity of a Si-capped Ge nanosheet on SOI compared to a GeOI nanosheet, measured between T = 3 K and 300 K. The substrate was grounded. While the GeOI nanosheet shows a distinct increase of the resistivity for lower temperatures, due to charge carrier freeze-out, the vertical Si-Ge-Si stack formed hole-gas, due to a confinement of holes close to the Ge-Si interface, revealing only a slight resistivity decrease.

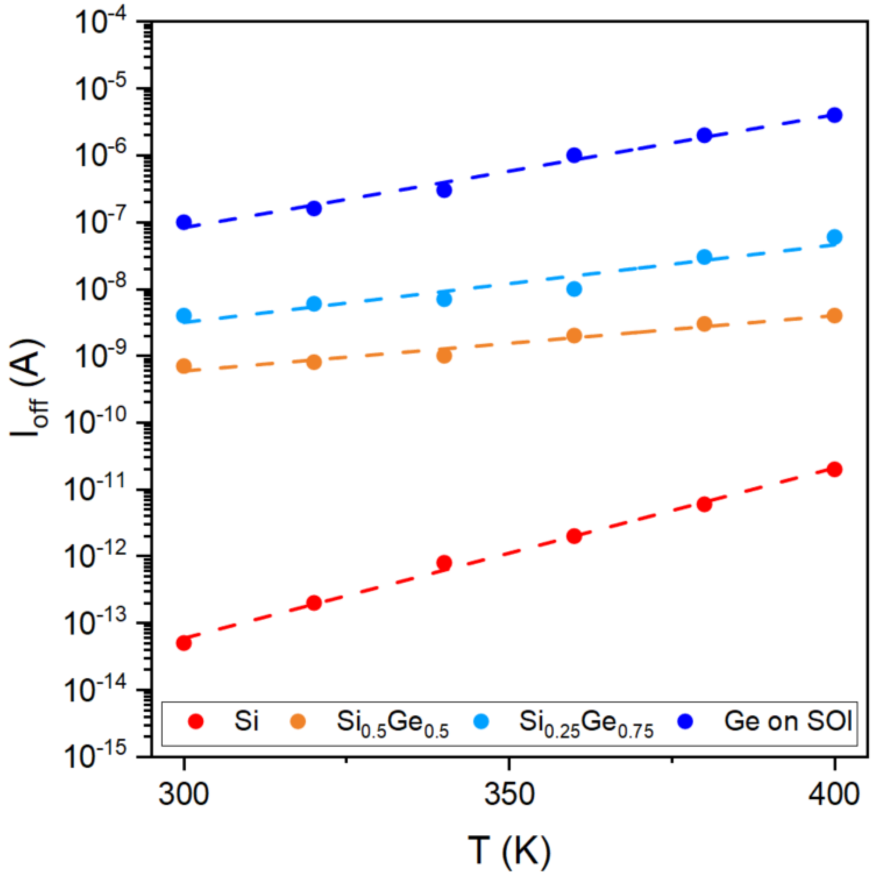

Figure S9: Temperature dependence of the off-current extracted from Figure 4 for all investigated $Si_{1-x}Ge_x$ compositions embedded in top-gated Al-$Si_{1-x}Ge_x$-Al heterostructures.

References

(1) Voort, G. F.; Asensio-Lozano, J. The Al-Si Phase Diagram. *Microscopy and Microanalysis* **2009**, *15*, 60–61.

(2) McAlister, A. J.; Murray, J. L. The Al-Ge (Aluminum-Germanium) system. *Bulletin of Alloy Phase Diagrams* **1984**, *5*, 341–347.

(3) Volin, T. E.; Balluffi, R. W. Annealing kinetics of Voids and the Self-diffusion Coefficient in Aluminum. *Physica Status Solidi (b)* **1968**, *25*, 163–173.

(4) Paccagnella, A.; Ottaviani, G.; Fabbri, P.; Ferla, G.; Queirolo, G. Silicon Diffusion in Aluminium. *Thin Solid Films* **1985**, *128*, 217–223.

(5) Beke, D. L. *Diffusion in Semiconductors*; Landolt-Börnstein - Group III Condensed Matter; Springer-Verlag: Berlin/Heidelberg, 1998; Vol. 33A.

(6) Gale, W.; Totemir, T. *Smithells Metals Reference Book*, 7th ed.; Reed Educational and Professional Publishing: Oxford, 1992.

(7) Trommer, J.; Heinzig, A.; Mühle, U.; Löffler, M.; Winzer, A.; Jordan, P. M.; Beister, J.; Baldauf, T.; Geidel, M.; Adolphi, B.; Zschech, E.; Mikolajick, T.; Weber, W. M. Enabling Energy Efficiency and Polarity Control in Germanium Nanowire Transistors by Individually Gated Nanojunctions. *ACS Nano* **2017**, *11*, 1704–1711.

(8) Xiang, J.; Lu, W.; Hu, Y.; Wu, Y.; Yan, H.; Lieber, C. M. Ge/Si nanowire heterostructures as high-performance field-effect transistors. *Nature* **2006**, *441*, 489–493.

(9) Rhoderick, E.; Williams, R. In *Metal-Semiconductor Contacts*, 2nd ed.; Hammond, P., Grimsdale, R., Eds.; Oxford University Press: Oxford, 1988; p 268.

(10) Thanailakis, A.; Chan, D. S.; Northrop, D. C. Activation Energy for the slow States in the Aluminium-Germanium Contact. *Journal of Physics D: Applied Physics* **1972**, *5*, 325.

(11) Pitale, S.; Ghosh, M.; Singh, S.; Manasawala, H.; Patra, G.; Sen, S. Characteristics of Al/Ge Schottky and ohmic Contacts at low Temperatures. *Materials Science in Semiconductor Processing* **2021**, *130*, 105820.
